# From incidentaloma to actionable insight: a clinical-molecular-imaging framework for risk-stratified management of testicular microlithiasis

**DOI:** 10.3389/fendo.2026.1803098

**Published:** 2026-04-15

**Authors:** Jiedong Zhou, Yong Ouyang, Shian Hu, Yuting Xiong, Min Liu

**Affiliations:** 1 The First Affiliated Hospital of Gannan Medical University Ganzhou, Jiangxi, China; 2 Department of Urology, The First Affiliated Hospital of Gannan Medical University Ganzhou, Jiangxi, China; 3 Gannan Medical University Ganzhou, Jiangxi, China; 4 Reproductive Medicine Department of the First Affiliated Hospital of Gannan Medical University Ganzhou, Jiangxi, China

**Keywords:** male infertility, risk stratification, scoping review, testicular dysgenesis syndrome, testicular germ cell tumor, testicular microlithiasis

## Abstract

**Objective:**

Testicular microlithiasis (TM) is common in infertile men, but its management is controversial due to an unclear link to testicular germ cell tumor (TGCT) risk. This scoping review synthesizes evidence to clarify the basis for personalized management of TM, focusing on infertile men.

**Methods:**

Following PRISMA-ScR guidelines, we systematically searched PubMed, Embase, and Web of Science (2015–2025) for studies on TM pathogenesis, imaging, molecular mechanisms, management, and TGCT association. Data were charted and narratively synthesized.

**Results:**

TM and TGCT share molecular pathways (e.g., KIT/KITLG, BMP7) within testicular dysgenesis syndrome. Isolated TM carries minimal risk, while coexisting with factors like cryptorchidism, infertility, or family history significantly elevates TGCT risk. Advanced imaging and liquid biopsy markers (e.g., miR-371a-3p) may refine risk assessment. Intermediate-risk patients (TM plus one established risk factor) may be considered for periodic ultrasound within shared decision-making; routine biomarker testing is not supported and should be individualized to selected high-risk contexts.

**Conclusion:**

The available evidence remains heterogeneous, and routine imaging surveillance for isolated testicular microlithiasis is not supported. We propose an evidence-informed, hypothesis-generating risk-stratification framework to support shared decision-making and highlight priorities for prospective validation.

## 1 Introduction

Testicular microlithiasis (TM) is a common incidental finding on scrotal ultrasonography, characterized by multiple punctate echogenic foci within the testicular parenchyma. Its clinical significance remains debated, primarily due to its uncertain association with testicular germ cell tumor (TGCT) and male infertility. Since the 1970s, the incidence of TGCT has steadily increased in developed regions such as Europe and North America, particularly among white men, although recent data suggest a plateau in some high-incidence countries (1). Globally, testicular cancer primarily affects young men aged 15–44 years, and overall case numbers are projected to continue rising (2). Despite this increasing incidence, early detection and the widespread use of platinum-based chemotherapy have significantly reduced mortality in high-income countries, with five-year survival rates remaining high (3). The rising incidence has been linked to socioeconomic development and may be associated with lifestyle-related factors in ecological and observational analyzes, including alcohol consumption, obesity, and physical inactivity, although causality remains uncertain (4). Given that TM is primarily detected by ultrasonography, understanding its radiological signatures and their clinicopathological correlates is essential. Despite frequent incidental detection on scrotal ultrasound, the clinical significance of TM remains debated, and follow-up practices vary widely across guidelines and clinical settings. Moreover, TM should be distinguished from scrotal calculi (also termed scrotal pearls), which are free-floating echogenic bodies within the tunica vaginalis cavity and represent fibrinous debris rather than intratubular calcifications; the two entities have distinct clinical implications and should not be conflated (5).

TM is characterized by multiple punctate echogenic foci within the testicular parenchyma, typically detected incidentally via ultrasound. Its etiology remains uncertain, and its clinical significance is primarily linked to male infertility and an increased risk of testicular cancer, although in most cases, it is considered a benign finding (6). According to the definitions provided by the European Association of Urology (EAU) and the European Society of Urogenital Radiology (ESUR), classic TM is defined as having ≥5 microliths per ultrasound field, whereas limited TM involves fewer than 5 microliths (7). With advancements in ultrasound resolution and the broader use of health screening, the prevalence of TM ranges from 0.7% to 6% in the general population but is significantly higher in high-risk groups, such as men with cryptorchidism, infertility, or disorders of sex development. Some studies report that TM prevalence reaches 11.2% within 10 years after orchiopexy for cryptorchidism and can exceed 20% in cases of high undescended testes (8).

Importantly, TM is frequently identified in men presenting with infertility, with prevalence substantially higher than in the general population. In infertile men, testicular microlithiasis has been reported to be associated with a substantially higher relative risk of testicular germ cell tumor in some cohorts (up to ~18-fold); however, estimates vary by study design and ascertainment, and absolute risk remains low in the absence of additional risk factors. The discovery of TM during a routine fertility work-up creates a management dilemma: balancing the need for oncological vigilance against the risk of causing undue patient anxiety and the burden of unnecessary surveillance. This underscores the urgent need for a clear, evidence-informed management pathway tailored to the reproductive medicine setting.

This uncertainty is reflected in, and perpetuated by, divergent recommendations in major urological guidelines. For instance, the European Association of Urology (EAU) guidelines do not recommend routine follow-up for isolated TM, whereas the European Society of Urogenital Radiology (ESUR) suggests considering annual ultrasound in high-risk settings, creating ambiguity in daily practice (9, 10). This lack of consensus leaves clinicians without a clear roadmap when TM is detected, often resulting in either unnecessary anxiety and overtreatment or, conversely, inadequate surveillance of high-risk individuals.

Earlier literature often regarded TM as an incidental finding that required no follow-up. However, since the late 1990s, an increasing number of studies have demonstrated an association between TM and TGCT, as well as germ cell neoplasia *in situ* (GCNIS), particularly when accompanied by other risk factors such as cryptorchidism, infertility, testicular atrophy, or a history of testicular cancer (11). A small number of observational analyses have explored whether TM co-occurrence in TGCT cohorts correlates with adverse oncologic outcomes; however, these findings are inconsistent and likely confounded (12).

This scoping review maps the multidisciplinary evidence linking TM, male infertility, and TGCT risk, and synthesizes areas of consensus and uncertainty across epidemiology, imaging, molecular studies, and clinical guidance. Rather than issuing prescriptive recommendations, we propose an evidence-informed framework to support shared decision-making and to identify research priorities for prospective validation.

**Figure 1:**
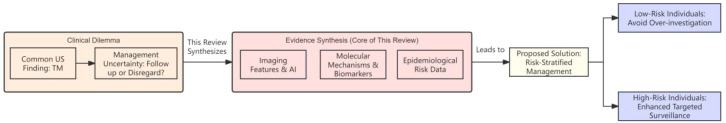
Bridging the clinical dilemma of TM through evidence synthesis. This scoping review addresses the uncertainty in managing incidentally discovered TM by systematically integrating key evidence from imaging, molecular studies, and epidemiology. The synthesis supports reframing management from a uniform approach to a personalized, risk-stratified strategy, aiming to reduce unnecessary anxiety and intervention in low-risk individuals while enabling targeted surveillance in high-risk groups.

Specifically, we aim to: (1) summarize mechanistic hypotheses connecting TM and TGCT within the testicular dysgenesis syndrome paradigm; (2) contextualize TM as a risk modifier rather than an isolated determinant; and (3) integrate guideline discrepancies into a pragmatic, risk-stratified approach aligned with screening-driven clinical realities in many Asian healthcare systems. The overall framework for evidence synthesis in this study is illustrated in **Figure 1**.

## 2 Methods

This study was designed as a scoping review to map and summarize the breadth of evidence on testicular microlithiasis and related follow-up practices, following the PRISMA-ScR reporting guideline. We restricted the primary search to 2015–2025 to capture contemporary evidence in the era of high-resolution ultrasound and modern TGCT biomarkers, while key earlier guidelines and landmark studies were additionally identified through hand-searching and citation tracking.

### 2.1 Identifying the research question

We aimed to map the breadth of evidence on TM management, focusing on its association with TGCT. Key questions included: (1) What are the pathogenic and molecular links? (2) What is the epidemiological evidence for TGCT risk, especially in high-risk populations? (3) What diagnostic and risk-assessment tools exist? (4) What risk-stratification models and management strategies are proposed?

### 2.2 Identifying relevant studies

A systematic search of PubMed, Embase, and Web of Science was performed from 2015 to 2025. Search terms included MeSH and keywords related to: TM, testicular neoplasms, germ cell tumor, ultrasonography, magnetic resonance imaging, biomarkers, risk assessment, and clinical management. The full PubMed strategy is in Supplementary Appendix 1. Reference lists of key reviews and guidelines were hand-searched.

### 2.3 Study selection

Inclusion Criteria: (1) Original observational/interventional studies on TM-TGCT association or management; (2) Systematic reviews/meta-analyses; (3) Clinical practice guidelines from major urological/radiological societies.

Exclusion Criteria: (1) Case reports/series (n<10); (2) Non-English publications; (3) Studies focusing solely on histopathology without clinical/imaging correlation; (4) Conference abstracts.

Two reviewers independently screened titles/abstracts and full texts. Discrepancies were resolved by discussion or a third reviewer.

### 2.4 Charting the data

Data were extracted into a standardized form: study characteristics, participant details, key findings on TM-TGCT association/diagnosis/management, and conclusions.

### 2.5 Collating and summarizing results

Given study heterogeneity, a narrative synthesis was performed. Evidence was collated into thematic areas emerging from the research questions.

Given the objectives of a scoping review, we did not perform a formal risk-of-bias assessment or grade the certainty of evidence. Instead, we focused on charting key study characteristics, populations, definitions of microlithiasis, and reported outcomes to identify areas of consistency, uncertainty, and evidence gaps.

## 3 Results

### 3.1 Study selection and characteristics of included evidence

**Figure 2:**
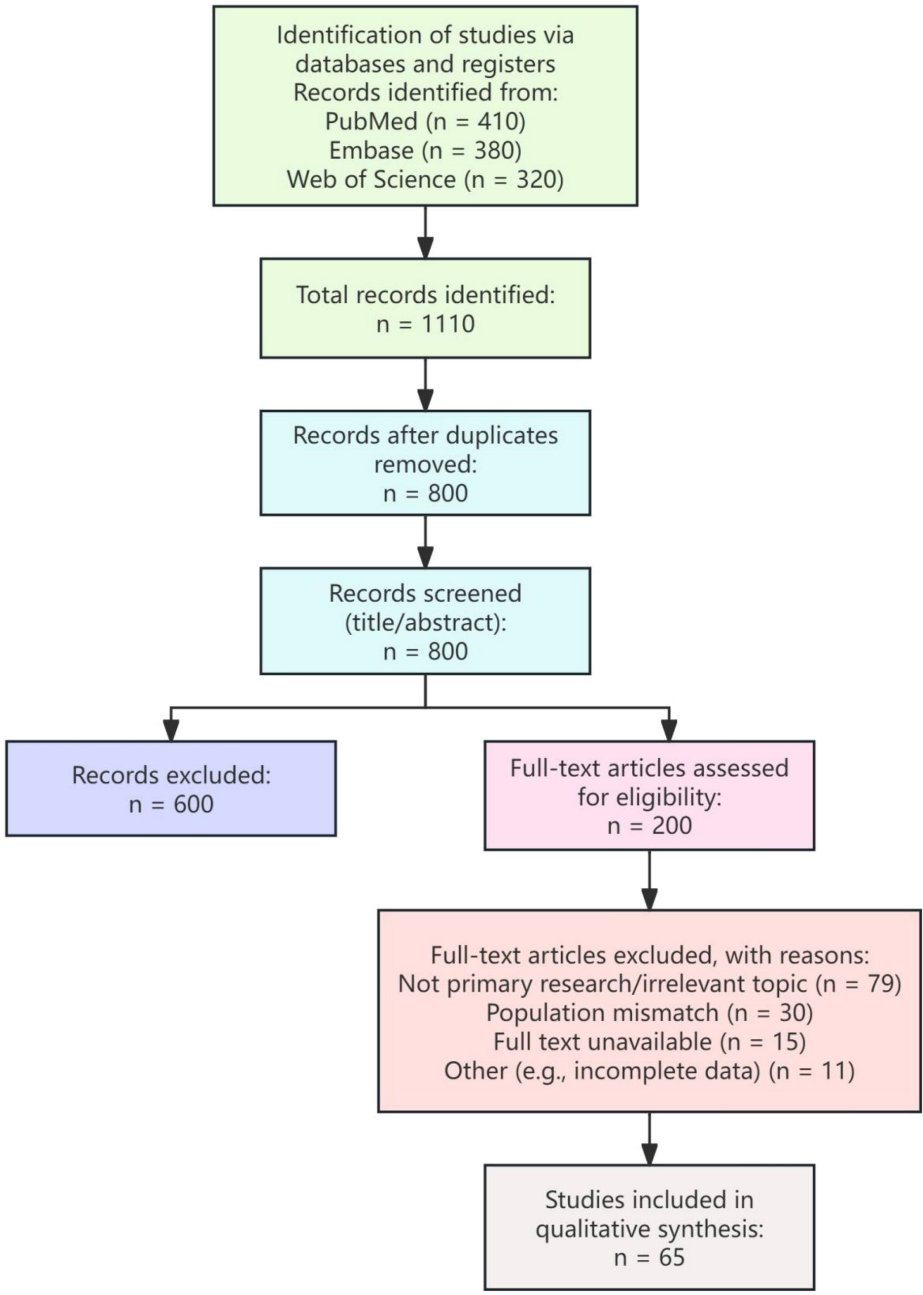
PRISMA flow diagram of the study selection process. This diagram illustrates the process of identifying, screening, and selecting studies for inclusion in this scoping review. The initial search across three databases (PubMed, Embase, Web of Science) yielded 1110 records. After removing 310 duplicates, 800 records underwent title/abstract screening. Following this, 200 full-text articles were assessed for eligibility. Ultimately, 65 studies met the predefined inclusion criteria and were included in the qualitative synthesis.

The study selection process is detailed in the PRISMA flow diagram (**Figure 2**). The 65 studies included in this synthesis encompassed a diverse range of evidence types, reflecting the multifaceted nature of the TM clinical dilemma. These comprised observational studies (e.g., cohort and case-control designs), systematic reviews and meta-analyses that quantitatively assessed the TM-TGCT association, clinical practice guidelines from major urological and radiological societies, and studies evaluating emerging diagnostic technologies (e.g., advanced imaging, artificial intelligence, liquid biopsy).

The publication timeline spanned from 2015 to 2025, indicating sustained and evolving research interest. Geographically, the evidence base was international, with contributions from North America, Europe, and Asia. This heterogeneity in study design and origin underscores the breadth of perspectives and the need for a scoping methodology to map the field. The following sections (3.2–3.6) present a narrative synthesis of this evidence, organized thematically from fundamental pathogenesis to clinical management strategies.

### 3.2 Pathogenesis and pathophysiological basis

#### 3.2.1 Formation of the TM

TM consists of laminated calcified bodies located within the seminiferous tubules, typically composed of a central organic core (such as cell debris or glycoproteins) surrounded by calcium phosphate deposits (13). Proposed mechanisms of TM formation include:

##### 3.2.1.1 Increased Germ Cell Apoptosis

Cellular remnants resulting from premature apoptosis caused by testicular injury, infection, ischemia, or genetic factors act as nuclei for calcification.

##### 3.2.1.2 Sertoli Cell Dysfunction

Sertoli cells typically clear apoptotic cells; impaired function results in the accumulation of cellular debris.

##### 3.2.1.3 Altered Tubular Microenvironment

Obstructed sperm transport or reduced fluid flow promotes calcium and phosphate precipitation, leading to laminated calcification (10). Intratubular microcalcifications correspond radiologically to the echogenic foci observed in classic TM on high-frequency ultrasonography.

#### 3.2.2 Molecular signaling pathways

Accumulating evidence suggests that TM and TGCT share molecular signaling abnormalities. Dysregulation of the KIT/KITLG pathway is considered a central driver, with mutations or aberrant activation forming the molecular basis for GCNIS and TGCT, particularly when accompanied by additional genetic or epigenetic alterations (14). Abnormal bone morphogenetic protein 7 (BMP7) signaling disrupts communication between Sertoli and Leydig cells, impairing spermatogenesis, facilitating microlith formation, and contributing to infertility (11). Imbalances in BCL2 antagonist/killer 1 (BAK1) further exacerbate germ cell apoptosis and debris accumulation, promoting calcification and increasing the risk of TM and TGCT (15). The accumulation of apoptotic debris and lipid-rich material within seminiferous tubules produces the highly reflective granular pattern seen on grayscale ultrasound.

#### 3.2.3 Testicular dysgenesis syndrome hypothesis

The TDS hypothesis offers an Integrative framework linking TM with TGCT. It proposes that fetal exposure to endocrine disruptors or genetic mutations results in abnormal development of Sertoli and Leydig cells, thereby increasing the risk of cryptorchidism, impaired spermatogenesis, TM, and TGCT in adulthood (16). Altered KIT/KITLG and BMP7 signaling contributes to aberrant germ cell clustering, which may manifest radiologically as focal heterogeneity or parenchymal texture alteration.

#### 3.2.4 Evidence from animal models

Animal studies provide direct support for the TDS hypothesis. Prenatal exposure to estrogens and phthalates has been shown to induce testicular dysgenesis, microlith formation, spermatogenic impairment, and GCNIS-like lesions in offspring (17). These findings support the causal link between fetal environmental or genetic insults and adult reproductive disorders, consistent with both epidemiological and molecular studies (18). Radiologically, oxidative stress–induced microvascular injury may explain the reduced vascular signal or perfusion heterogeneity occasionally detected on color Doppler and contrast-enhanced ultrasonography.

#### 3.2.5 Summary

**Figure 3:**
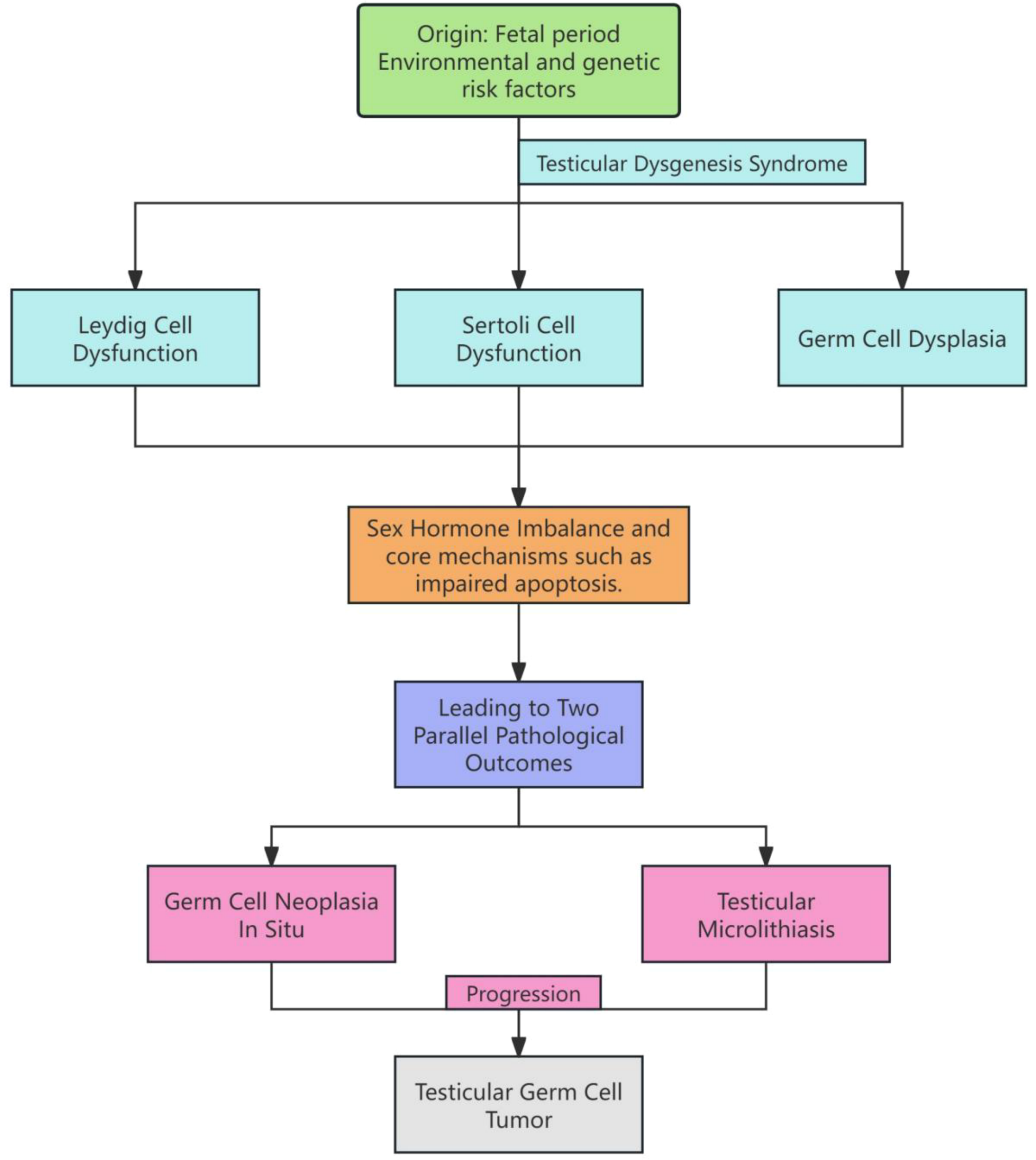
Shared pathophysiological pathways: linking testicular dysgenesis to microlithiasis and germ cell tumorigenesis. Fetal environmental exposures or genetic factors may contribute to TDS, leading to dysfunction of Leydig and Sertoli cells, as well as abnormal germ cell development. These changes, through mechanisms such as hormonal imbalances and impaired apoptosis, can result in two parallel pathological outcomes: the formation of TM and the development of GCNIS, which may ultimately progress to invasive testicular germ cell tumors.

In summary, the development of TM is driven by multiple interacting factors. Increased germ cell apoptosis, impaired Sertoli cell function, and alterations in the intratubular microenvironment provide the direct pathological basis for the formation of calcified cores. Additionally, abnormalities in key signaling pathways such as KIT/KITLG, BMP7, and BAK1 suggest that TM and TGCT may share overlapping molecular mechanisms. Furthermore, the TDS hypothesis offers a developmental perspective, linking fetal environmental exposures or genetic mutations to the occurrence of TM, TGCT, and reproductive dysfunction in adulthood. Evidence from animal models directly supports this hypothesis, reinforcing the causal relationship between abnormal testicular development and subsequent disease. Taken together, these findings indicate that TM should not be regarded as a mere incidental imaging finding but rather as a reflection of deeper pathophysiological disturbances in testicular development and function. This integrated understanding also provides important insights into its clinical significance and association with TGCT. To better illustrate the potential pathophysiological links between TM and TGCT as outlined by the TDS hypothesis, we have integrated these mechanisms into the following schematic diagram (**Figure 3**): Pathophysiological Model of TM and TGCT Development. This mechanistic overlap not only explains the frequent co-occurrence of TM and TGCT but also provides a molecular rationale for why TM serves as a sensitive, albeit non-specific, biomarker of underlying testicular dysregulation and oncogenic potential.

### 3.3 Epidemiology and high-risk populations

#### 3.3.1 General population

TM is relatively uncommon in the general male population and is usually detected incidentally during health check-ups or reproductive screenings. In the general population, TM is usually detected incidentally and appears to confer little additional TGCT risk in the absence of other risk factors (11).

#### 3.3.2 Special populations

In certain populations, the prevalence of TM is significantly higher, and the risk of TGCT is correspondingly increased.

##### 3.3.2.1 Cryptorchidism

The risk of TGCT is increased 4–8-fold. Elevated testicular temperature and impaired spermatogenesis may promote microlith formation and malignant transformation (19).

##### 3.3.2.2 Infertile men

In infertile men, TM has been associated with a markedly increased relative risk of TGCT in some cohorts (reported up to ~18-fold), although estimates vary by study design, follow-up duration, and ascertainment; the absolute risk remains low without additional risk factors (20).

##### 3.3.2.3 Chromosomal abnormalities

The prevalence of TM exceeds 20% in patients with Klinefelter syndrome, suggesting a link between chromosomal abnormalities, testicular dysgenesis, and increased cancer risk (21).

##### 3.3.2.4 Positive family history

Men with a first-degree relative affected by TGCT face a 3- to 10-fold increased risk, which is further amplified in the presence of TM (22).

#### 3.3.3 Evidence from meta-analyses

Systematic reviews and meta-analyses have consistently substantiated the association between TM and TGCT, particularly within high-risk subgroups. Quantitative syntheses of large cohorts demonstrate that TM significantly elevates the risk of TGCT, with the magnitude of risk varying considerably based on the presence of concurrent factors. Specifically, the coexistence of TM with conditions such as male infertility, cryptorchidism, or a positive family history has been shown to confer a substantially higher risk compared to isolated TM. These findings collectively underscore that TM functions not merely as an incidental imaging finding but as a contextual marker of TGCT susceptibility, especially in the presence of other established risk factors (23, 24).

#### 3.3.4 Clinical significance and risk stratification

Isolated TM is usually benign; however, in high-risk populations, it significantly increases TGCT risk (25). Based on the coexistence of TM with other risk factors to guide individualized follow-up and early detection strategies. Future research should focus on multicenter longitudinal cohorts that integrate imaging with molecular biomarkers to optimize early detection and prevention of TGCT.

### 3.4 Diagnosis and imaging characteristics

#### 3.4.1 Ultrasound classification

Evidence indicates that classic TM, defined as ≥5 microliths per ultrasound field, is more strongly associated with TGCT, particularly in patients with additional risk factors such as cryptorchidism, infertility, or testicular atrophy, where the risk of malignancy is significantly increased (10, 26, 27). Limited TM (<5 microliths) generally carries a lower risk; however, careful follow-up is warranted when it is accompanied by other abnormal ultrasound findings or clinical risk factors (28). Recent multicenter cohort studies suggest that combining TM classification with clinical risk factors helps optimize the frequency and scope of follow-up, enabling individualized precision management and improving early detection and intervention in high-risk patients (6, 29).

#### 3.4.2 Associated ultrasound findings

Patients with TM often present with reduced testicular volume, heterogeneous parenchymal echotexture, or focal hypoechoic lesions, all of which indicate an increased risk of malignancy (30). A comprehensive risk assessment that integrates ultrasound features, clinical history, and high-risk factors enhances the sensitivity of TGCT detection (31). Some studies suggest that quantitative ultrasound measures—such as the proportion of hypoechoic areas within the testicular volume and changes in vascular signals—may serve as potential markers to guide follow-up and intervention (32).

#### 3.4.3 Advanced imaging technologies

Emerging imaging modalities demonstrate potential value in the diagnosis and risk stratification of TM.

##### 3.4.3.1 Shear-wave elastography

Provides quantitative assessment of testicular tissue stiffness, significantly improving early identification and differential diagnosis of suspicious lesions. Combined use with conventional ultrasound is recommended to enhance clinical decision-making (33, 34).

##### 3.4.3.2 Magnetic resonance imaging

MRI is useful in cases where ultrasound evaluation is inconclusive or when invasive lesions are suspected. It clearly delineates lesion boundaries and tissue characteristics, thereby improving diagnostic accuracy and tumor staging (35, 36).

##### 3.4.3.3 Artificial intelligence-assisted diagnosis

Deep learning–based algorithms can automatically detect TM, quantify calcification features, and integrate risk factors to predict malignancy risk, offering new avenues for large-scale screening and personalized management (37). AI-assisted tools may improve TM detection, microlith burden quantification, and integration of imaging phenotypes with clinical risk factors for individualized risk stratification; however, TM-specific prospective validation is currently lacking (38).

### 3.5 Evidence for the association between TM and testicular cancer

#### 3.5.1 Imaging modalities in testicular assessment

Ultrasound remains the first-line imaging technique for TM detection due to its accessibility, safety, and cost-effectiveness. However, novel modalities such as contrast-enhanced ultrasound (CEUS), shear-wave elastography (SWE), multiparametric MRI, and radiomics-based artificial intelligence (AI) are increasingly being incorporated into urological practice. Understanding their technical principles and diagnostic utilities is essential for optimizing patient management.

#### 3.5.2 Studies in adults

Multiple cohort studies have demonstrated a statistically significant association between TM and TGCT, particularly in high-risk populations.

Large-scale observational studies conducted over the past decade have consistently indicated that isolated TM is associated with a modest increase in relative risk for TGCT, though the absolute risk remains low in asymptomatic individuals without additional risk factors. This pattern underscores the importance of contextualizing TM within the broader clinical profile rather than interpreting it as an independent high-risk marker (39).

In a long-term, single-center follow-up study, Patel et al. (2016) observed 442 patients with TM for up to 14 years. Only two cases (0.5%) of TGCT were detected, both occurring in individuals with additional risk factors such as cryptorchidism, infertility, or a family history of testicular cancer. This finding indicates that isolated TM confers minimal malignant potential, whereas the risk is significantly higher when other risk modifiers are present (40).

Guidelines and expert consensus in the field consistently highlight that TM should not be viewed as an independent risk factor for TGCT. Current evidence suggests that routine imaging surveillance in asymptomatic patients with isolated TM offers limited clinical benefit. However, when TM coexists with established risk factors such as cryptorchidism, infertility, or a family history of TGCT, it serves as a significant clinical indicator, warranting heightened vigilance and further evaluation within a risk-stratified management framework (41).

#### 3.5.3 Studies in children and adolescents

The clinical significance of TM in pediatric and adolescent populations remains controversial (10).

##### 3.5.3.1 Minimal risk with isolated TM

The review highlighted that TM alone does not constitute a direct risk factor for testicular cancer in this age group; therefore, invasive investigations or interventions are generally unnecessary.

##### 3.5.3.2 High-risk co-factors as key determinants

When TM coexists with cryptorchidism, testicular atrophy, or a family history of testicular cancer, the malignant potential increases significantly, necessitating close surveillance or early intervention.

##### 3.5.3.3 Individualized management

Recent literature suggests that children with isolated TM generally require reassurance, education, and periodic clinical examination rather than routine imaging surveillance; those with additional risk factors may be considered for periodic ultrasound monitoring (42).

#### 3.5.4 Histological and pathological evidence

Histopathological analyses of TGCT resection specimens have revealed TM in approximately 40–50% of cases, with an even higher prevalence in patients with GCNIS (43). This suggests that TM may not be merely an incidental ultrasonographic finding but could reflect tumor-related microenvironmental alterations. Studies have demonstrated that seminiferous tubules adjacent to TM often exhibit basement membrane thickening, stromal fibrosis, and infiltration by immune cells such as lymphocytes and macrophages (44). These pathological changes may lead to local ischemia, chronic inflammation, and microenvironmental remodeling, thereby promoting the survival and proliferation of abnormal germ cells (45). Moreover, molecular pathology studies have shown elevated Ki-67 expression and activation of the p53 signaling pathway in regions surrounding TM, indicating a potential link to early carcinogenesis (46). While these findings do not conclusively establish a direct oncogenic role for TM, the strong association with GCNIS and local microenvironmental abnormalities suggests that TM may serve as a “warning signal” warranting attention in clinical surveillance and risk stratification (13, 47).

#### 3.5.5 TM as a risk modifier: moving beyond the ‘independent risk factor’ debate

Despite frequent observations of TM in epidemiological and pathological studies, whether it constitutes an independent risk factor for TGCT remains highly controversial. Large cohort follow-up studies have shown that the incidence of TGCT in patients with isolated TM is comparable to that in the general population. However, when TM coexists with cryptorchidism, a family history of TGCT, or a previous history of TGCT, the risk of malignancy increases substantially (10, 12). This underscores that TM should not be evaluated in isolation but rather within the context of each patient’s full clinical background, with stratified management strategies accordingly (48). Nevertheless, some scholars argue that even in the absence of overt high-risk features, TM may indicate subtle microenvironmental abnormalities and advocate for more intensive follow-up. Overall, the current consensus favors considering TM as a risk modifier rather than an independent risk factor, with its primary clinical value lying in prompting clinicians to identify and manage concomitant risk factors. Prospective large-scale studies integrating histological and molecular evidence are urgently needed to clarify the precise role of TM in TGCT initiation and progression, thereby informing individualized surveillance strategies (49).

### 3.6 Risk stratification and management strategies

**Figure 4:**
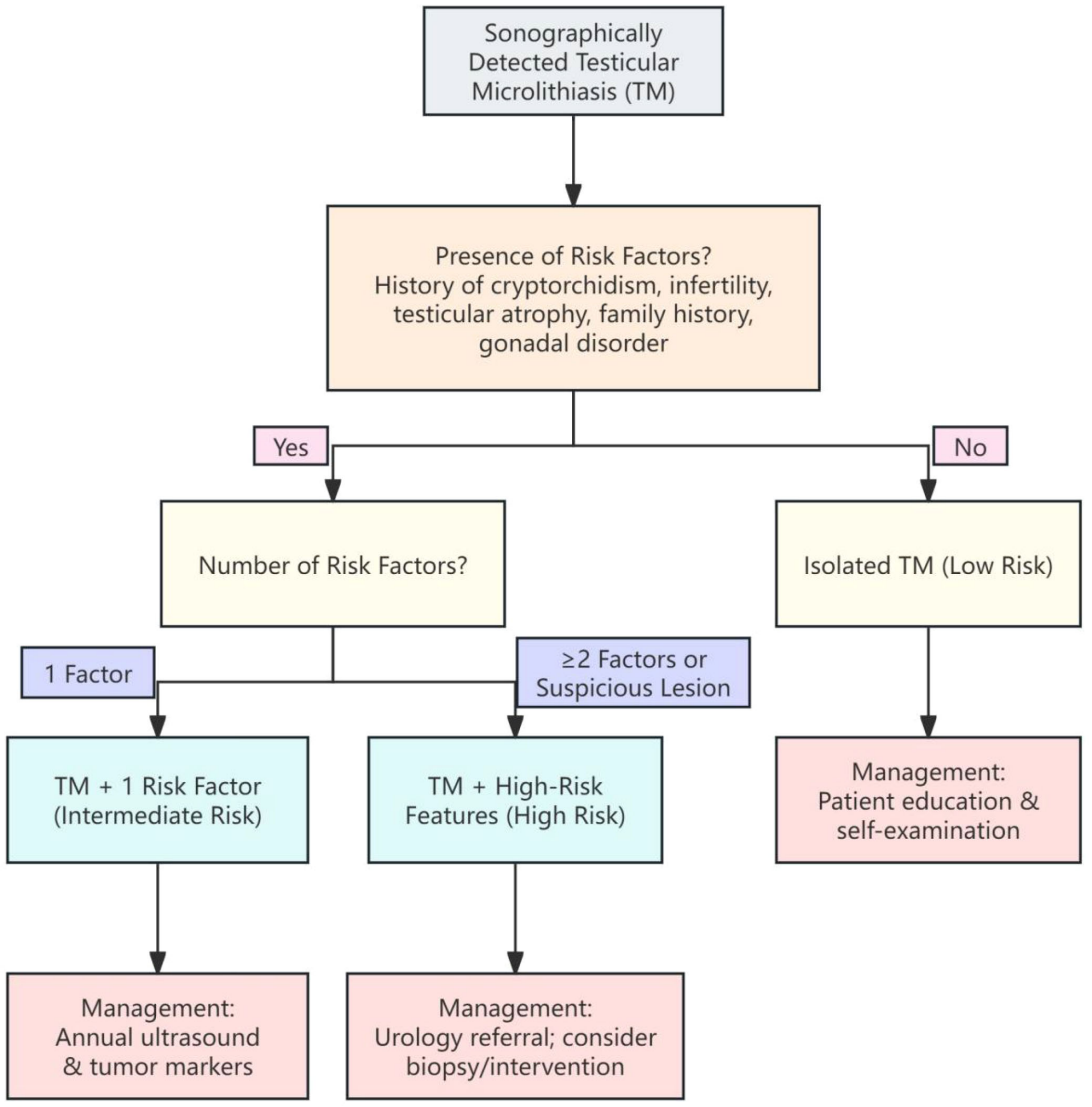
Clinical decision algorithm for the risk-stratified management of TM. This figure presents a proposed evidence-informed framework (not a clinical guideline) intended to support shared decision-making; prospective validation is required before routine implementation. This flowchart provides a stepwise approach for managing patients with incidentally detected TM. Management intensity is stratified based on the presence and number of established clinical risk factors.

Based on the synthesis above, we propose an integrated, risk-stratified management framework (**Figure 4**). This framework is not a clinical guideline and requires prospective validation.

#### 3.6.1 Risk stratification models

Polygenic risk scores (PRS) have been proposed to refine TGCT susceptibility stratification; however, their clinical utility in TM cohorts remains unvalidated and currently investigational. Therefore, PRS is discussed here as a potential future adjunct rather than a routine clinical tool. Risk stratification and corresponding management recommendations for patients with TM are presented in **Table 1** (50).

**Table 1:** Impact of different risk factor combinations on TGCT risk and clinical recommendations.

Risk factor combination	Change in TGCT risk	Clinical recommendation
TM alone	Slightly increased	No routine imaging
TM + cryptorchidism/family history/high PRS	Significantly increased (6–8-fold), intensified surveillance	Consider periodic ultrasound within shared decision-making; biomarker testing should be individualized in selected high-risk contexts (e.g., investigational use of miR-371a-3p).
High-risk occupation/environmental exposure	Increased	Individualized risk assessment

PRS, polygenic risk score. “Increased risk” estimates are primarily derived from epidemiological and meta-analytic studies. Consider periodic ultrasound; biomarker testing should be individualized in selected high-risk contexts (e.g., research use of miR-371a-3p).

#### 3.6.2 International guideline recommendations

A summary of international guideline recommendations for the management and follow-up of testicular microlithiasis is presented in **Table 2**.

**Table 2:** Summary of international guideline recommendations for TM management and follow-up.

Guideline	Low-risk (isolated TM)	High-risk (with additional risk factors)	Follow-up duration
EAU 2023	No follow-up required	Annual ultrasound + self-examination	Until age 55
AUA 2021	Not recommended	Consider surveillance in the presence of additional risk factors (details individualized)	Individualized
NICE 2020	No follow-up	Annual screening in high-risk groups	Continuous
ESUR 2015	Self-examination advised	Annual ultrasound (high-risk); ± tumor markers in selected contexts	Continuous

Some guideline statements are derived from earlier versions and expert consensus; recommendations on serum tumor markers apply only to selected high-risk contexts and should not be interpreted as routine testing for asymptomatic isolated TM. EAU, European Association of Urology; AUA, American Urological Association; NICE, National Institute for Health and Care Excellence; ESUR, European Society of Urogenital Radiology; Recommendations reflect the latest available guidelines.

#### 3.6.3 Proposed integrated follow-up strategy

Based on a synthesis of guidelines from the EAU, ESUR, AUA, and NICE, as well as findings from meta-analyses and risk stratification models, the following surveillance recommendations are proposed:

##### 3.6.3.1 Low-risk (isolated testicular microlithiasis)

For individuals with isolated microlithiasis and no additional risk factors (e.g., history of cryptorchidism, prior germ cell tumor, testicular atrophy, infertility, or a first-degree family history), routine ultrasound surveillance is not recommended. Management should emphasize reassurance, patient education, and regular testicular self-examination, with clinical review and repeat imaging reserved for new symptoms, palpable abnormalities, or evolving risk profiles (51).

##### 3.6.3.2 Intermediate risk (TM plus a single high-risk factor)

For individuals with microlithiasis and additional risk factors, annual ultrasound may be considered as part of a shared decision-making approach; however, evidence supporting routine serum tumor marker testing in asymptomatic patients without a mass is limited, and its use should be individualized or confined to selected high-risk contexts. MRI may be considered when necessary. A multimodal assessment approach enhances early detection of TGCT and optimizes patient management (52, 53).

##### 3.6.3.3 High risk (≥2 risk factors or suspicious lesions)

Referral to urology for long-term management is recommended. In selected cases with a suspicious focal lesion or discordant findings, further evaluation (e.g., targeted imaging, specialist review, and consideration of biopsy according to institutional practice) may be warranted (49).

## 4 Discussion

### 4.1 Principal findings and clinical translation

This scoping review synthesized evidence from 65 studies to map the current landscape of TM management. The principal finding is that the clinical significance of TM is not intrinsic but context-dependent. Isolated TM carries minimal risk, comparable to that of the general population, whereas its coexistence with established risk factors (e.g., cryptorchidism, infertility, family history) multiplicatively increases TGCT risk. This evidence supports a paradigm shift from viewing TM as an independent risk factor to regarding it as a risk modifier and a biomarker of underlying testicular dysgenesis. For clinical practice, this underscores that the discovery of TM should not provoke anxiety but should prompt a systematic clinical evaluation to identify and contextualize these concomitant risk factors.

### 4.2 Key controversies: causality and population specificity

#### 4.2.1 Uncertain causality

The synthesized evidence highlights a fundamental controversy: association does not imply causation. Most studies are cross-sectional or retrospective, limiting causal inference (54). While TM frequently coexists with GCNIS and shares molecular pathways (e.g., KIT/KITLG, BMP7) with TGCT within the TDS framework (55, 56). This does not establish TM as a direct precursor. It is more likely that both conditions arise from shared developmental or genetic insults (25). This uncertainty is central to the divergent management guidelines.

#### 4.2.2 Differences between pediatric and adult populations

The clinical implications of TM differ significantly by age. In children and adolescents, evidence suggests a weak association with future TGCT, with risk potentially increasing only after puberty. For children with isolated TM, most evidence supports reassurance, education, and periodic clinical examination rather than routine imaging surveillance (57). This contrasts with adults, where the risk is better defined and stratified management is more clearly warranted (7).

### 4.3 Future directions: integrating novel tools into risk-stratified care

#### 4.3.1 The promise and challenge of AI and advanced imaging

Emerging approaches such as radiomics, machine learning, and circulating biomarkers may enable more individualized risk assessment in the future; however, current evidence is largely exploratory and not yet specific to risk prediction in testicular microlithiasis cohorts (58, 59). Future studies should prioritize prospective, well-phenotyped microlithiasis populations with standardized definitions, clinically meaningful outcomes, and external validation.

#### 4.3.2 Liquid biopsy and biomarker integration

Circulating miR-371a-3p has emerged as a superior biomarker for the diagnosis and monitoring of TGCT compared to traditional markers such as AFP, hCG, and LDH. Its future application may involve a “dual-channel” screening model that combines imaging with liquid biopsy to enable dynamic risk assessment in high-risk TM patients (60, 61). Validating this approach in TM-specific cohorts is a critical next step.

#### 4.3.3 The imperative for higher-level evidence

A significant gap identified by this review is the near-total absence of multicenter randomized controlled trials (RCTs) evaluating TM surveillance strategies. Future research must prioritize RCTs that compare different follow-up intensities (e.g., ultrasound intervals, biomarker testing) to assess outcomes such as TGCT detection rates, patient anxiety, quality of life, and cost-effectiveness. Such studies are essential to transition from expert opinion to evidence-informed standards (62).

### 4.4 Beyond oncology: implications for reproductive health and fertility preservation

TM, especially when diagnosed in infertile men, offers a crucial insight into overall testicular health. The shared etiology among TM, impaired spermatogenesis, and TGCT suggests an underlying testicular dysgenesis syndrome (63). Therefore, the clinical evaluation of TM should include an assessment of reproductive potential. For high-risk individuals, counseling should incorporate discussions on fertility preservation strategies, such as semen cryopreservation, thereby aligning oncological vigilance with reproductive life planning and respecting patient autonomy (64).

### 4.5 Limitations of the evidence and this review

The limitations of the primary evidence, which is predominantly retrospective and observational, have been acknowledged. As a scoping review, this study has inherent limitations: it aimed to map the breadth of evidence rather than to appraise the quality of individual studies in depth, a task more appropriate for a systematic review. Although our search was systematic across three major databases, restricting inclusion to English-language publications may have introduced selection bias. Nonetheless, this approach effectively provides a comprehensive overview of the current knowledge landscape and its gaps.

The proposed framework is derived predominantly from observational, retrospective evidence, which constitutes its primary limitation. While it offers a rational and evidence-informed approach, its recommendations—particularly regarding the frequency and modality of surveillance in intermediate-risk groups—require validation in prospective, preferably multicenter, studies. Future research should also incorporate cost-effectiveness analyses to ensure the framework’s sustainability in diverse healthcare economies.

### 4.6 Considerations in Asian clinical practice

The risk-stratified framework proposed herein is particularly relevant to clinical practice in many Asian healthcare systems. Notably, the widespread adoption of comprehensive annual health check-ups—including scrotal ultrasonography—in countries such as Japan, South Korea, and China results in a higher frequency of incidentally detected TM. This screening-driven context underscores the need for clear management algorithms to prevent over-investigation of low-risk findings. Additionally, cultural factors may influence patient anxiety and adherence to long-term surveillance. Our proposed strategy, which emphasizes patient education and self-examination for low-risk cases alongside clear pathways for higher-risk individuals, aligns with the demands of both proactive screening environments and resource-conscious settings common in Asia. Future studies validating this framework in Asian cohorts will be invaluable.

## 5 Conclusion

The synthesized evidence may help contextualize existing guideline statements. The risk-stratified framework presented here is intended as an evidence-informed supplement to support shared decision-making and to guide priorities for prospective validation, rather than as a prescriptive guideline.

TM epitomizes the convergence of developmental biology, environmental exposure, and imaging science within urology. Moving beyond the binary question of “Is TM premalignant?”, we advocate for a stratified approach that utilizes TM as a context-dependent biomarker. The integration of AI-quantified imaging phenotypes with circulating molecular markers (e.g., miR-371a-3p) may enable more dynamic risk assessment in the future. Future efforts must prioritize the prospective validation of these tools and the development of evidence-informed, cost-effective surveillance protocols that reduce patient anxiety while protecting those at genuine risk. By doing so, TM transforms from a clinical dilemma to a strategic asset in the early interception of TGCT.
